# Tryptophan hydroxylase 2 deficiency alters autism-related behavioural phenotypes in rats

**DOI:** 10.1038/s41598-025-05684-9

**Published:** 2025-07-01

**Authors:** Joanna Golebiowska, Małgorzata Holuj, Natalia Alenina, Michael Bader, Piotr Popik, Agnieszka Nikiforuk

**Affiliations:** 1 Department of Behavioral Neuroscience and Drug Development, Maj Institute of Pharmacology, Polish Academy of Sciences, 12 Smetna Street, 31-343 Kraków, Poland; 2 Max-Delbrück-Center for Molecular Medicine in the Helmholtz Association (MDC), Berlin, Germany; 3 German Center for Cardiovascular Research (DZHK), Partner Site Berlin, Berlin, Germany; 4 Experimental and Clinical Research Center, a cooperation between the Max-Delbrück-Center for Molecular Medicine in the Helmholtz Association and the Charité University Medicine, Berlin, Germany; 5 Institute for Biology, University of Lübeck, Lübeck, Germany

**Keywords:** TPH2-KO, Autism, USV, Rats, Serotonin, 5-HT, Autism spectrum disorders, Developmental disorders, Social behaviour, Neuroscience

## Abstract

Serotonin is a crucial neurotransmitter implicated in various physiological processes. Dysregulation of the serotonergic system, particularly in early life, may be associated with neuropsychiatric disorders, including autism spectrum disorder. This study utilises a rat model lacking the central serotonin-synthesising enzyme tryptophan hydroxylase 2 (TPH2) to investigate the effects of brain serotonin depletion on core autism-related behaviours. The animals’ social behaviour was assessed using social interaction and resident-intruder tests, along with ultrasonic vocalisation analysis to evaluate communication deficits. Repetitive behaviours were examined through the marble burying test and the attentional set-shifting task to measure cognitive flexibility. Our findings indicate that TPH2-deficient (TPH2-KO) rats displayed altered patterns of social behaviour and vocalisation. Notably, the study highlights sex differences in the behavioural responses of TPH2-KO rats, with males showing increased aggression and copulation–like behaviour. Additionally, male TPH2-KO rats demonstrated cognitive inflexibility, while females displayed significant compulsivity in marble-burying tests. This research highlights the serotonergic system’s role in socio-communicative and repetitive behaviours, establishing the TPH2-KO rat model as a valuable tool for exploring the neurobiological underpinnings of autism and related disorders. Further investigations into the differential effects of serotonin depletion across sexes may enhance our understanding of the mechanisms underlying neuropsychiatric conditions.

**Supplementary Information:**

The online version contains supplementary material available at 10.1038/s41598-025-05684-9.

## Introduction

Serotonin (5-hydroxytryptamine, 5-HT) is a multi-functional molecule extensively distributed throughout the body. It functions as an autacoid in the periphery and as a neurotransmitter and trophic factor in the adult and developing nervous systems. As a neurotransmitter, it is implicated in a broad range of physiological processes, including regulation of sleep/wakefulness, temperature, nociception, mood, stress, aggression, and sexual and maternal behaviours^1^. As a trophic factor, serotonin regulates cell proliferation, differentiation and migration, making it essential for neurodevelopmental processes^2,3^. Disturbances in the serotonergic system, particularly those occurring early in life, can contribute to the development of a wide range of neuropsychiatric disorders, such as autism spectrum disorder (ASD), depression, anxiety, obsessive-compulsive disorder and schizophrenia^2,4^.

Serotonin synthesis is controlled by tryptophan hydroxylase (TPH), the rate-limiting enzyme in the serotonin production pathway. There are two isoforms of the TPH, TPH1 and TPH2, each encoded by distinct genes in mammals^5^. TPH1 primarily regulates peripheral serotonin synthesis in the gut, while TPH2 is responsible for serotonin production in the brain. These two systems are functionally distinct, not only because different genes encode the two TPH isoenzymes but also due to serotonin’s inability to cross the blood-brain barrier^5^. Thus, disruptions in central serotonin synthesis through TPH2 dysfunctions, which specifically inactivate serotonin production in the brain^6,7^, are particularly relevant for understanding the mechanisms underlying neurodevelopmental disorders^8^.

Multiple lines of evidence suggest a link between dysregulation of the serotonergic system and autism pathology^3,9–11^. In particular, an association between polymorphisms in the *TPH2* gene and the development of ASD was demonstrated in humans^12,13^. Mice lacking brain serotonin exhibited some autistic traits^14,15^. Moreover, the enhancement of serotonergic signalling has been recently shown to improve social deficits in mouse models of ASD^16,17^, further confirming the critical role of the serotonergic system in autism pathology. However, understanding the mechanisms underlying these interactions is still elusive, and further evaluation of preclinical serotonergic models is essential for developing and validating novel therapeutics for ASD.

ASD is a lifelong neurodevelopmental condition that typically emerges in early childhood. Its core symptoms include social and communication deficits, along with repetitive behaviours^18^. In preclinical studies, a range of behavioural tests may be used to quantify autism-like symptoms in animal models. The social interaction test, where two unfamiliar rodents are allowed to interact freely, provides a reliable method for assessing social functions^14,19,20^. The resident-intruder paradigm provides a valuable method for evaluating the aggressive components of social behaviour^21^. This is particularly relevant for low-brain serotonin models, which tend to exhibit heightened aggression^22–24^. Rodents communicate with each other through ultrasonic vocalisations (USVs). The USVs of adult rats can be divided into 50-kHz and 22-kHz frequency calls, depending on the emotional state in which they are emitted^25,26^. The first group, 50-kHz calls, are typically produced in appetitive situations, particularly during social interactions^27^ and reward anticipation^28,29^. The second group, often referred to as ’alarm’ calls, consists of 22-kHz calls, which are emitted during aversive states, such as in the presence of danger, predators, or aggressive conspecifics, as well as during fighting or fear-related behaviours^25,26^. Examination of USVs emitted during social interaction and the resident-intruder tests may provide critical insights into communication impairments. Notably, the analysis of frequency-based categorisations of USVs in rats offers a nuanced approach that is highly relevant for evaluating animal models of ASD^25,26,30^. Repetitive behaviour, the final component of the triad of core symptoms of ASD, can be categorised into two types: motor actions, characterised by repetitive movements, and behaviours involving a cognitive component, such as cognitive rigidity^31^. In rodents, motor behaviour is often assessed using the marble burying test, which measures repetitive digging behaviour^32,33^. Conversely, the attentional set-shifting task (ASST) is used to evaluate the degree of cognitive inflexibility^34^.

Although deficits in social communication are a core feature of ASD, they remain poorly characterised in models based on TPH2 deficiency. To address this underexplored aspect, the primary aim of this study was to evaluate ultrasonic vocalisations in a rat model lacking brain serotonin due to the congenital lack of TPH2 (TPH2-KO)^35^. These animals exhibit undetectable levels of serotonin and its metabolite 5-HIAA in relevant brain areas and display heightened aggression along with symptoms of impulse control and anxiety disorders^36,37^. TPH2-KO rats are considered a valuable model for studying neurodevelopmental disorders, as they mimic the impact of serotonin deficiency on neuronal plasticity during early brain development^38^.

While many prior studies in this field have utilised mice, we selected rats for several reasons. First, rats exhibit a more complex and nuanced social behaviour repertoire, including a richer range of vocal communication^39–43^. These characteristics make rats particularly advantageous for studying ASD, where socio-communicative deficits represent the most prominent and critical symptoms. Moreover, the high baseline levels of aggression observed in mice complicate the interpretation of social interaction measures and often preclude the controlled assessment of affiliative behaviours^44^. Finally, rats’ complex cognitive abilities make them especially suitable for studying higher-order cognitive functions, such as those assessed using the ASST^42,43^.

Therefore, alongside social behaviour assessment, USVs emitted by TPH2-KO and wild-type (WT) rats during social interaction and resident-intruder tests were analysed to identify potential abnormalities in social communication. Moreover, both motor and cognitive forms of restricted, repetitive behaviours were evaluated using the marble burying test and the ASST, respectively.

Sex differences may affect susceptibility to psychiatric disorders associated with altered brain serotonin concentrations, such as autism. Regulation within the serotonergic system, including serotonin synthesis, levels of its metabolites, transporter activity and receptor expression, may vary according to sex^38^. Hence, we used both male and female mutant rats to investigate potential sex differences in animal behaviour.

## Methods

### Animals

The rats (Max Delbrück Center for Molecular Medicine, Berlin) were 3 months old at the beginning of the experiments. They were housed in a temperature-controlled (21 ± 1 °C) and humidity-controlled (40–50%) colony room under a 12/12 h light/dark cycle (lights on at 06:00 h). They were group-housed (4 rats/cage) with free access to food and water. Behavioural testing was performed during the light phase of the light/dark cycle. The experiments were conducted in accordance with the European Guidelines for Animal Welfare (2010/63/E.U.) and were approved by the II Local Ethics Committee for Animal Experiments at the Maj Institute of Pharmacology, Polish Academy of Science, Krakow, Poland (permission number: 92/2017). This study was performed in accordance with the ARRIVE guidelines.

Adult TPH2-deficient (TPH2-KO) and wild-type (TPH2-WT) rats on the Dark Agouti (DA) background^35^ were bred at the Max Delbrück Center for Molecular Medicine (Berlin) by crossing animals heterozygous for the TPH2 mutation and genotyped as previously described^45^. The TPH2-KO rats carry a deletion in exon 7 of the rat *Tph2* gene, resulting in a frame shift and a premature stop in translation. The resulting homozygous animals completely lack serotonin in the brain from birth throughout their whole life^35^. Previous studies have shown that the DA strain is suitable for use in behavioural research^46^.

The total number of animals used in this study was *N* = 19 (TPH2-WT males), *N* = 40 (TPH2-WT females), *N* = 21 (TPH2-KO males), and *N* = 41 (TPH2-KO females). We included a higher proportion of females than males while acknowledging potential variability due to the oestrous cycle. This approach aimed to ensure adequate female representation in our study while accounting for possible hormonal influences on experimental outcomes. All animals (3 months old) were initially subjected to the social interaction test. For subsequent behavioural assessments, randomly selected animals from the same cohort were consecutively subjected to the marble burying, resident-intruder, and attentional set-shifting tasks. Randomisation accounted for each animal’s prior testing history to ensure unbiased allocation and to avoid overburdening individual animals. A minimum of one week was allowed between consecutive tests to reduce potential carry-over effects.

### Social interaction test

The social interaction test between same-sex and same-genotype pairs of rats was performed as previously reported^40,47^. The experiments were conducted in an open-field arena (57 × 67 × 30 cm) made of black Plexiglas with a grey floor. The arena was dimly illuminated with an indirect light of 15 lx. One day before the test, the animals were handled and weighed, and on the test day, half of the animals had their backs marked with acrylic paint (A’kryl Renesans). To acclimate animals to the testing area, each was individually placed in the open field for 5 min^40,47^. On the test day, two unfamiliar rats of matched body weight (± 5 g) were placed in the open-field arena, and their behaviours were recorded for 10 min using a Sony light-amplification CCD camera positioned above the arena and connected to a PC running a Noldus MPEG recorder 2.1. An experimenter, blind to the treatment conditions, manually analyzed the videos offline using Noldus Observer^®^ XT (version 10.5).

The measured social behaviours included sniffing (the rat sniffs the body of the conspecific), anogenital sniffing (the rat sniffs the conspecific’s anogenital region), social grooming (the rat licks and chews the conspecific’s fur), following (the rat moves toward and follows the conspecific), and climbing (the rat climbs over or stands on the conspecific’s back). Additionally, we scored aggressive (fighting) and copulatory-like behaviours^40,47^. The number of episodes of social behaviour was measured for each rat separately and then summed to give a total score for each pair of animals. In cases where the total number of animals in a group was odd (e.g., *N* = 19 for TPH2-WT males and *N* = 21 for TPH2-KO males), one animal was paired twice with different partners to ensure a sufficient number of pairs for meaningful statistical analysis. Additionally, two TPH2-KO female pairs were excluded from the final analysis due to technical issues with the video recordings that made behavioural scoring unreliable. Consequently, the number of pairs analysed was as follows: *N* = 10 (TPH2-WT males), *N* = 20 (TPH2-WT females), *N* = 11 (TPH2-KO males), and *N* = 18 (TPH2-KO females).

### Resident-intruder test

The procedure was adapted from the classic resident–intruder test^21^ and conducted as previously described^20^. The test took place in the home cages of singly housed resident rats, with a camera (E314351, Basler, Ahrensburg, Germany) positioned above the testing area and connected to a PC running Noldus Observer^®^ XT, version 10.5. The testing area was dimly illuminated by the light of 18 lx.

One day before the test, resident rats (TPH2-WT and TPH2-KO rats of both sexes) were individually housed with ad libitum access to food and water. Naive intruder rats (TPH2-WT) were used in the interaction with the residents only once. On the testing day, the rats were moved to the experimental room for a 30-minute acclimatisation period. The test began by introducing an unfamiliar, same-sex, slightly smaller (approximately 1–25 g) intruder into the resident’s home cage. Interactions between the resident and the intruder were recorded for 10 min^20^. After the test completion, both rats were returned to their respective group cages. The behaviours of each resident rat were scored manually using Noldus Observer^®^ XT by an experimenter blinded to the treatment conditions. Behaviours were classified as either dominant (lateral threatening, sideway lateral pushing, clinch attacking, chasing, standing on top of the supine intruder, aggressive grooming, boxing, biting) or submissive (supine postures, fleeing behaviour, defensive upright posture, freezing).

The number of pairs (i.e., resident + intruder) used in the analysis was *N* = 18 (TPH2-WT males), *N* = 30 (TPH2-WT females), *N* = 17 (TPH2-KO males), and *N* = 30 (TPH2-KO females).

### USVs recording

As previously described^48^, the rats’ vocalisations were recorded using a microphone with a frequency response range of 2–200 kHz (UltraSoundGate Condensor Microphone CM16/CMPA, Avisoft Bioacoustics, Berlin, Germany) positioned 25 cm above the test area floor (social interaction test and resident intruder test). The microphone signals were transmitted to an UltraSoundGate 416 H (Avisoft Bioacoustics, Berlin, Germany) before the analogue signal was digitised with a sampling rate of 200 kHz and a 16-bit resolution. Acoustic data were recorded using Raven Pro: Interactive Sound Analysis Software, version 1.5 (The Cornell Lab of Ornithology Bioacoustics Research Program, Ithaca, NY, USA). Using Raven Pro software (version 1.5), calls were manually marked on the computer screen by an experienced user blind to the treatment. The spectrograms were generated with a fast Fourier transform (FFT) with a length of 512 points and a 75% time-window overlap (100% frame, Hamming window).

The USV analysis included the evaluation of 50-kHz USVs along with the following USV features: (a) the number of USVs, (b) call duration (length of the call, measured in milliseconds), (c) bandwidth (the difference between the highest and lowest frequencies, reflecting frequency modulation, expressed in kHz), and (d) peak frequency (the frequency in kHz where the highest energy occurs). Additionally, we manually divided the calls, based on their acoustic call features, into the following general types: short calls, flat calls with a near-constant frequency, and frequency-modulated calls. The frequency-modulated included mostly trills but also one-component calls (complex, ramp, and inverted-U calls) and multi-component calls (multi-step, step-up, step-down, and composite calls). We also analysed the number of 22-kHz USVs. The sample size (N) for the USV analysis was the same as that employed in the social interaction and resident-intruder experiments, as specified in the preceding sections.

### Marble burying test

Clean cages (27 × 16.5 × 12.5 cm) were filled with a 4 cm layer of chipped wood bedding. Twenty-five green glass marbles (20 mm diameter) were gently placed on top of the bedding in a 5 × 5 grid, equidistant from each other. Animals were introduced into the testing cage, and the number of marbles buried (defined as more than 50% covered by bedding) over 30 min was recorded. Additionally, the distance travelled was automatically measured using the Any-maze^®^ tracking system.

The number of animals in a given group was *N* = 16 (TPH2-WT males), *N* = 19 (TPH2-WT females), *N* = 15 (TPH2-KO males), and *N* = 20 (TPH2-KO females).

### Attentional set shifting task (ASST)

As previously described^34^, testing was conducted in a dimly illuminated (20 Lux) Plexiglas apparatus (length x width x height: 38 × 38 × 17 cm) with a grid floor and a wall dividing half of the length of the cage into two sections. During testing, one glass-digging pot was placed in each section. Each pot was defined by a pair of stimulus cues, incorporating two dimensions: odour and digging medium. However, only one pot (the ’positive’ pot) was baited with a food reward (half of a Honey Nut Cheerio, Nestle^®^), which was buried at the bottom of the pot beneath the digging medium.

The procedure was carried out over three consecutive days for each rat. On the first day, the rats were habituated to the testing area and trained to dig in pots filled with sawdust to retrieve a food reward. On the second day, rats were trained on a series of simple discriminations (SDs) to a criterion of six consecutive correct trials. In these trials, the rats had to learn to associate the food reward with either an odour cue or a digging medium. On the final day, the rats performed a series of 7 discriminations in a single test session: simple discrimination (SD), compound discrimination (CD), reversal 1 (Rev1), intradimensional shift (ID), reversal 2 (Rev 2), extradimensional shift (ED), and reversal 3 (Rev 3). Testing in each phase continued until the rat met the criterion of six consecutive correct trials to criterion (TTC), after which the test proceeded to the next phase. A detailed task description is provided in Supplement 1.

The number of animals in a given group was *N* = 9 (TPH2-WT males), *N* = 17 (TPH2-WT females), *N* = 11 (TPH2-KO males), and *N* = 13 (TPH2-KO females).

### Identification of the oestrous cycle phase

After each test, vaginal cytology samples were collected, and the stage of the oestrous cycle was determined by examining the appearance and abundance of cells in the vaginal samples, as previously described in detail by^20^. Further details are provided in Supplement 2.

### Statistics

We used a priori planned comparisons to assess genotype effects within each sex. In addition, two-way ANOVAs with genotype (TPH2-WT vs. TPH2-KO) and sex (male vs. female) as between-subject factors were conducted to examine overall main effects and interactions. For the social interaction and resident-intruder tests, different types of behaviours assessed were analyzed as within-subject (repeated measures) factors. For the attentional set-shifting task, the different task stages (SD, CD, IDS, EDS, etc.) were treated as repeated measures. When applicable, sphericity was assessed using Mauchly’s test, and when the assumption of sphericity was violated, the Greenhouse–Geisser correction was applied (i.e., SI, ASST). When a significant main effect of genotype was found, Tukey HSD post hoc tests were used to assess overall differences between genotypes. Effect sizes were estimated using partial eta squared (ŋ2). The normality of data distribution was evaluated by the Kolmogorov-Smirnov test. The sample size was determined using G*Power software (version 3.1.9.4), based on an alpha level of 0.05 and a statistical power of 85–90%.

Statistical significance was set at *p* < 0.05. All statistical analyses were performed using Statistica 12.0 for Windows. Detailed ANOVA results are presented in Supplement 3.

## Results

### TPH2 deficiency differentially affects social interaction patterns in male and female rats

**Fig. 1:**
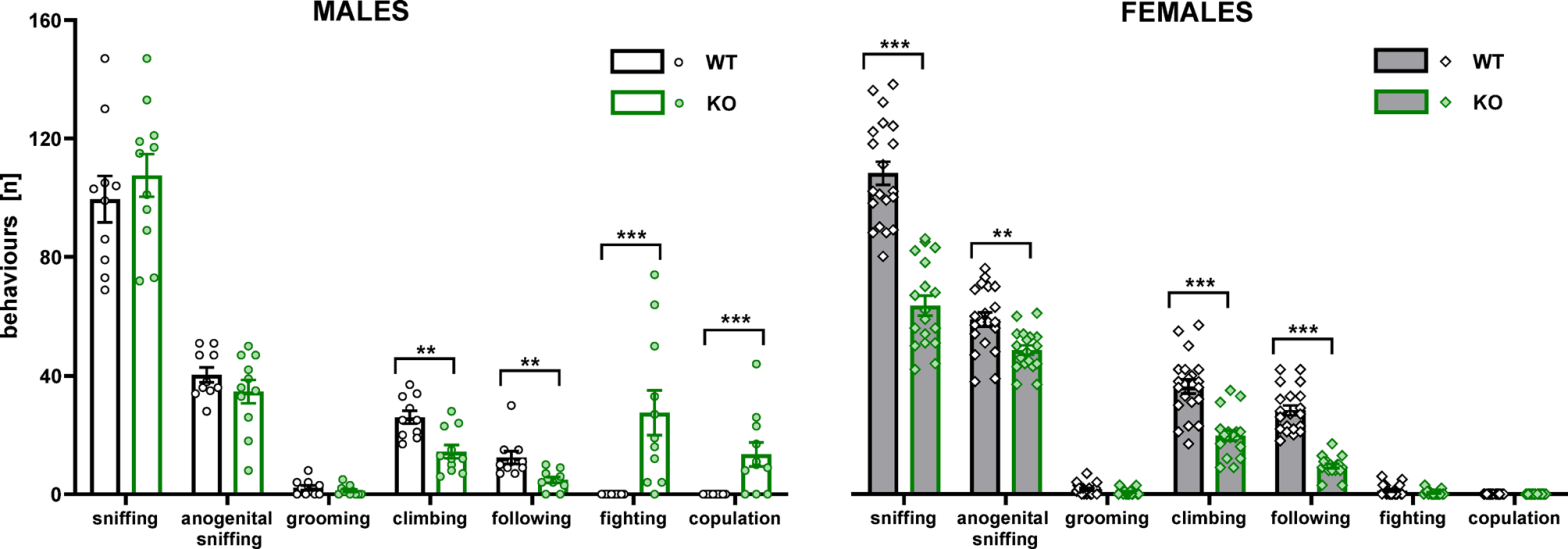
TPH2 deletion affects social behaviour patterns. Male (left panel) and female (right panel) TPH2-WT and TPH2-KO rats were subjected to a social interaction test with a partner of the same sex and genotype. The following behaviours were analysed: sniffing, anogenital sniffing, grooming, climbing, following, fighting, and copulatory-like behaviours. Data are presented as mean ± SEM of the number of episodes of each behaviour. Symbols: **p < 0.01, ***p < 0.001 a significant difference between TPH2-WT and TPH2-KO animals in a given sex (planned comparisons).

As illustrated in Fig. 1, the pattern of social behaviours differed between TPH2-KO and WT rats in a sex-specific manner (genotype × sex × behaviour interaction: F[2.75,151.37] = 11.48, *p* < 0.001, Table S.3.1 Supplement 3). Planned comparisons revealed that pairs of TPH2-KO males demonstrated an increase in aggressive (t = 5.84, *p* < 0.001) and copulatory-like (t = 5.38, *p* < 0.001) behaviours in comparison to WT males. In contrast, pairs of TPH2-KO females displayed a significant reduction in sniffing (t = 7.09, *p* < 0.001), anogenital sniffing (t = 3.24, *p* < 0.01), climbing (t = 5.91, *p* < 0.001) and following (t = 10.28, *p* < 0.001) behaviours in comparison to WT females.

The impact of the oestrous cycle on social behaviour did not differ between TPH2-WT or TPH2-KO females (Fig. S.2.1. Supplement 2).

### Lack of TPH2 affects social interaction-induced communication in a sex-specific manner

**Fig. 2:**
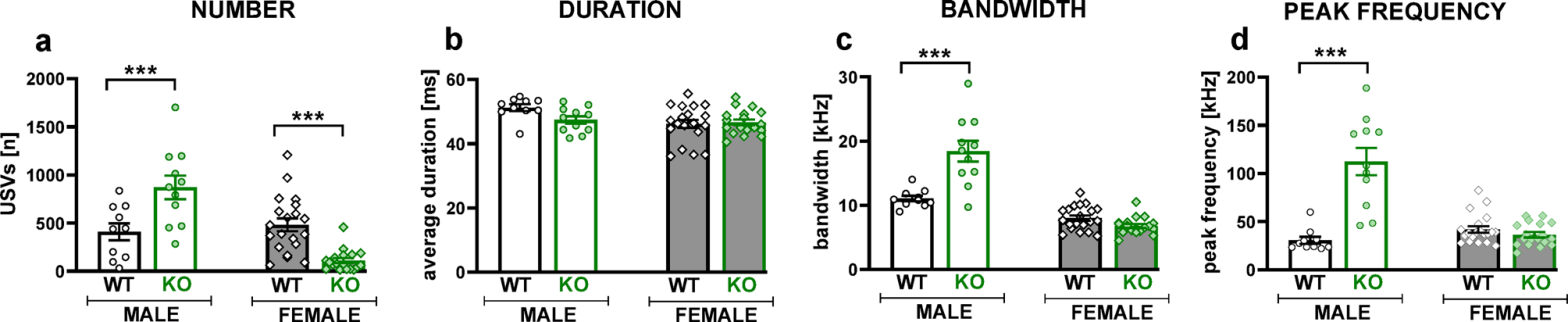
TPH2 deletion affects USV emission during social interaction in a sex-dependent manner. USV recordings were conducted during 10 minutes of the social interaction test in male and female TPH2-WT and TPH2-KO rats with a partner of the same sex and genotype. Data are presented as mean ± SEM of the (**a**) total number, (**b**) average duration, (**c**) bandwidth, and (**d**) peak frequency of 50-kHz calls. Symbols: ***p < 0.001, a significant difference between TPH2-WT and TPH2-KO animals in a given sex (planned comparisons).

TPH2 deletion also affected 50-kHz USV emission during social interactions, and these effects were also sex-specific (genotype x sex interactions for the USV number: F[1,55] = 30.52, *p* < 0.001, bandwidth: F[1,55] = 34.37, *p* < 0.001 and peak frequency: F[1,55] = 48.03, *p* < 0.001; Fig. 2, Table S.3.2 Supplement 3). TPH2-KO males emitted significantly more USVs compared to the controls (t = 3.81, *p* < 0.001; Fig. 2a). Their calls were characterised by a wider bandwidth (t = 6.27, *p* < 0.001; Fig. 2c) and a higher peak frequency (t = 8.09, *p* < 0.001; Fig. 2d). On the contrary, a reduced number of USVs was demonstrated in TPH2-KO females (t = 4.13, *p* < 0.001; Fig. 2a); however, their calls did not differ from controls in any of the acoustic parameters measured (Fig. 2b–d).

The relation of 22-kHz emitting to non-emitting rats did not differ between groups (males: TPH2-WT: 7/3 and TPH2-KO: 7/4, Chi^2^ = 0.024, NS; females: TPH2-WT: 5/15 and TPH2-KO: 4/14, Chi^2^ = 0.032, NS). TPH2-KO rats did not differ from their wild-type controls in terms of the number of emitted 22-kHz calls (males: TPH2-WT: 146.9 ± 61.94 and TPH2-KO: 143.4 ± 42.69; females: TPH2-WT: 8.8 ± 3.8 and TPH2-KO: 2.0 ± 0.4; Fig S.4.1a Supplement 4).

#### Call type characteristics

There were no significant effects of TPH2 deletion on the distribution of 50-kHz call categories (trills, one-component, multi-component, short and flat calls; Fig S.4.2a, Table S.4.1 Supplement 4).

#### Correlations

**Fig. 3:**
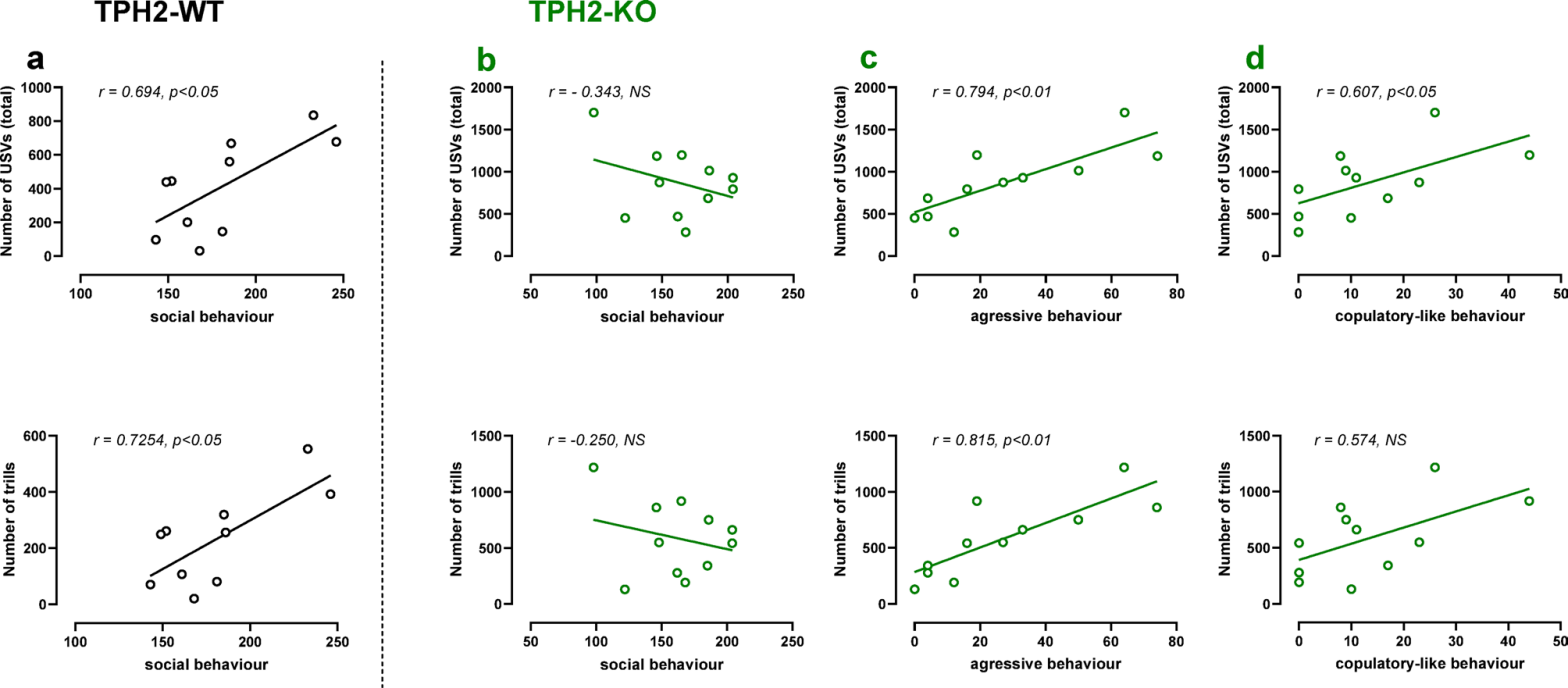
Correlation analysis between behavioural patterns and 50-kHz USV emission in male rats. A Pearson correlation coefficient (r) was calculated to examine the correlation between the total number of social (**a**, **b**), aggressive (**c**) or copulation-like (**d**) behaviours and the total number of 50-kHz USVs (upper panel) or trills (lower panel) during social interaction in WT (**a**) and TPH2-KO (**b**, **c**, **d**) male rats.

To examine the relationship between social behaviours and USV emission, correlation analyses were conducted (Fig. 3). In TPH2-WT males, a positive correlation was found between the total number of 50-kHz emitted calls and the total episodes of social behaviours (*r* = 0.694, *p* < 0.05) but no such correlation was observed in TPH2-KO males (*r* = -0.343, NS). However, in TPH2-KO males, the total number of USVs was positively correlated with fighting (*r* = 0.794, *p* < 0.01) and copulatory-like (*r* = 0.607, *p* < 0.05) behaviours.

Further analyses of specific call categories in TPH2-WT males revealed a positive correlation between social behaviours and trills (*r* = 0.7254, *p* < 0.05; Fig. 3). In contrast, in TPH2-KO males, trills were positively correlated with aggressive behaviours (*r* = 0.815, *p* < 0.01; Fig. 3).

No significant correlation was found between social behaviours and the total number of USVs or trills in TPH2-WT females (data not shown).

### TPH2 deficiency increases aggression and dominant behaviour

**Fig. 4:**
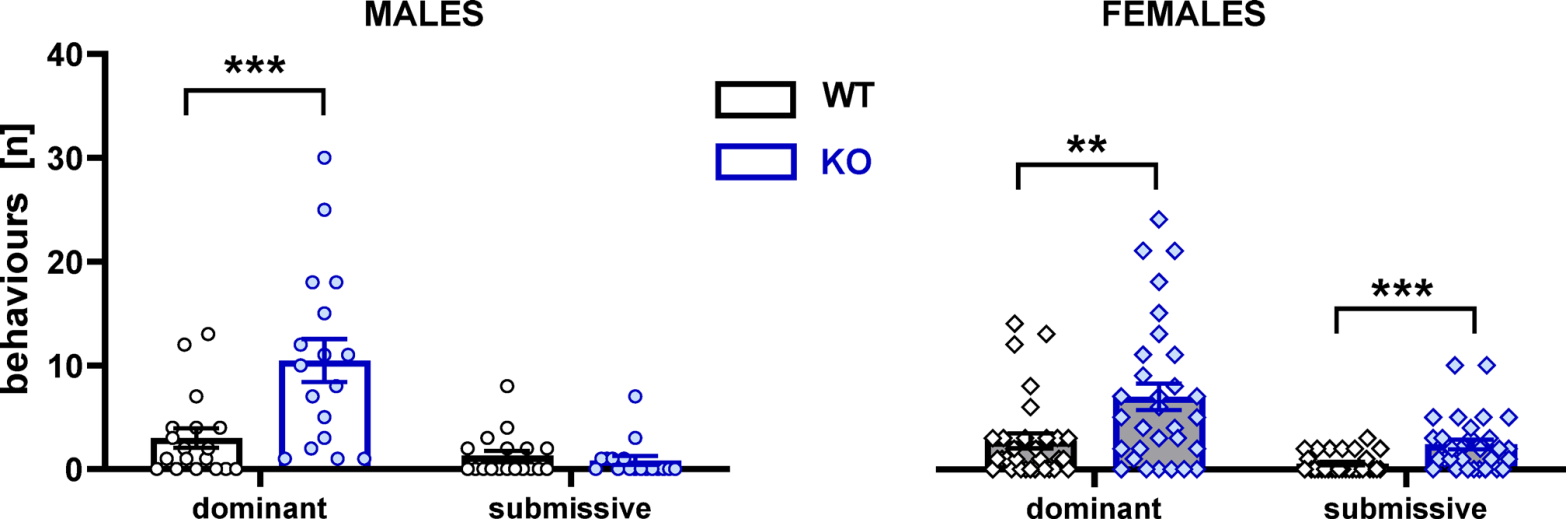
TPH2 deletion affects the behavioural repertoire in response to the presence of an intruder. Dominant and submissive behaviours were analysed during 10 min of the resident-intruder test with male (left panel) and female (right panel) TPH2-WT and TPH2-KO resident rats using a naive, unfamiliar wild-type intruder of the same sex. Data are presented as mean ± SEM of the number of episodes of dominant and submissive behaviour. Symbols: **p < 0.01, ***p < 0.001 a significant difference between TPH2-WT and TPH2-KO animals in a given sex (planned comparisons).

TPH2 deletion also affected the behaviour of rats during resident-intruder encounters (genotype × sex × behaviour interaction: F[1,91] = 4.75, *p* < 0.05; Fig. 4, Table S.3.3. Supplement 3). Planned comparisons revealed that TPH2-KO males and females exhibited more dominance episodes than their wild-type controls (males: t = 3.67, *p* < 0.001 and females: t = 2.73, *p* < 0.01; Fig. 4). Moreover, submissive behaviours were increased in TPH2-KO females (t = 3.59, *p* < 0.001; Fig. 4).

The oestrous cycle did not affect female residents’ behaviour (Fig.S.2.2, Table S.2.3. Supplement 2).

### TPH2 deficiency affects resident-intruder encounter-induced USVs in a sex-specific manner

**Fig. 5:**
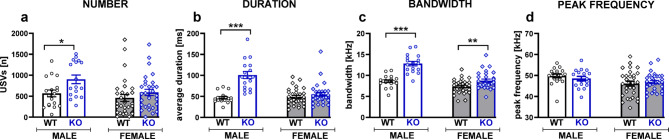
TPH2 deletion affects the vocalisation pattern during intruder encounters. USV recordings were conducted during 10 minutes of the resident-intruder test with male and female TPH2-WT and TPH2-KO resident rats, using a naive, unfamiliar wild-type intruder of the same sex. Data are presented as mean ± SEM of the (**a**) total number, (**b**) average duration, (**c**) bandwidth, and (**d**) peak frequency of 50-kHz calls. Symbols: *p < 0.05, **p < 0.01, ***p < 0.001, a significant difference between TPH2-WT and TPH2-KO animals in a given sex (planned comparisons).

TPH2 deletion also had significant effects on the number of USVs emitted during the resident-intruder test (genotype effect: F[1,91] = 7.1, *p* < 0.01; Fig. 5a, Table S.3.4 Supplement 3) and on the acoustic features of calls, i.e., duration (genotype effect: F[1,91] = 34.13, *p* < 0.001; Fig. 5b, Table S.3.4 Supplement 3) and bandwidth (genotype effect: F[1,91] = 50.48, *p* < 0.001; Fig. 5c, Table S.3.4 Supplement 3), but not peak frequency (Fig. 5d, Table S.3.4 Supplement 3). Planned comparisons revealed that pairs including TPH2-KO males as residents emitted more USVs (t = 2.41, *p* < 0.05), and their calls were longer (t = 6.62, *p* < 0.001) and had a broader bandwidth (t = 6.69, *p* < 0.001) than those emitted by control pairs. Between-genotype differences in females’ calls were restricted to the wider bandwidth in TPH2-KO females (t = 2.94, *p* < 0.05; Fig. 5a, b, c).

Significantly more TPH2-KO males emitted 22-kHz calls compared to TPH2-WT males (relation of alarm-emitting to non-emitting rats: TPH2-WT: 3/15 and TPH2-KO: 13/4, Chi2 = 12.6, *p* < 0.001). No differences were demonstrated for females (TPH2-WT: 7/23 and TPH2-KO: 7/23). The average number of 22-kHz calls in USV-emitting animals was higher for TPH2-KO animals (males: TPH2-WT: 70.0 ± 34.3 and TPH2-KO: 110.8 ± 27.4; females: TPH2-WT: 9.4 ± 2.8 and TPH2-KO: 69.3 ± 42.4; Fig. S.4.1b Supplement 4).

#### Call type characteristics

TPH2 deletion influenced the distribution of 50-kHz call categories in a sex-dependent manner (call type × genotype × sex: F[1.65,149.83] = 8.01, *p* < 0.01; Fig.S.4.2.b, Table S.4.2 Supplement 4). Planned comparisons revealed that pairs in which a TPH2-KO male rat was a resident emitted more trills (t = 8.17, *p* < 0.001) and fewer one-component calls (t = 4.07, *p* < 0.001) than those including wild-type controls as residents (Fig. S.4.2b Supplement 4).

### TPH2 deficiency enhances repetitive behaviour in the marble burying test

**Fig. 6:**
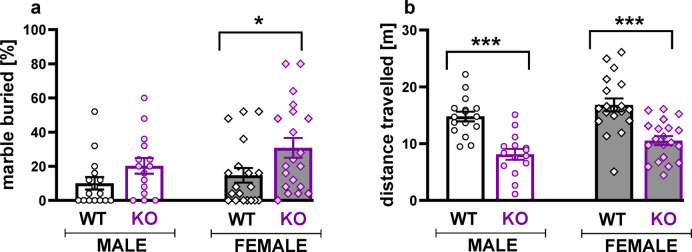
TPH2 deletion increases marble burying activity. The percentage of buried marbles (**a**) and distance travelled (**b**) were quantified for male and female TPH2-WT and TPH2-KO rats after 30 min of the test. Data are presented as mean ± SEM. Symbols: *p < 0.05, ***p < 0.001 a significant difference between TPH2-WT and TPH2-KO animals in a given sex (planned comparisons).

TPH2 deletion significantly increased the number of buried marbles (*p* < 0.01, Tukey HSD post hoc test following a significant genotype effect: F[1,66] = 7.43, *p* < 0.01; Fig. 6a, Table S.3.5 Supplement 3). Planned comparisons revealed that TPH2-KO females buried more marbles than their WT counterparts (t = 2.49, *p* < 0.05).

The increased marble-burying behaviour did not appear to stem from heightened locomotor activity, as TPH2-KO animals showed a reduction in the distance travelled during the test (*p* < 0.001, Tukey HSD post hoc test following a significant genotype effect: F[1,66] = 45.28, *p* < 0.001; planned comparisons: males: t = 4.65, *p* < 0.001, females: t = 4.89, *p* < 0.001; Fig. 6b).

We did not observe any effects of the oestrous cycle on marble burring or locomotor activity in either TPH2-WT or TPH2-KO females (Fig. S.2.3, Table S.2.4 Supplement 2).

### **TPH2 deficiency results in cognitive inflexibility in the attentional set-shifting task**

**Fig. 7:**
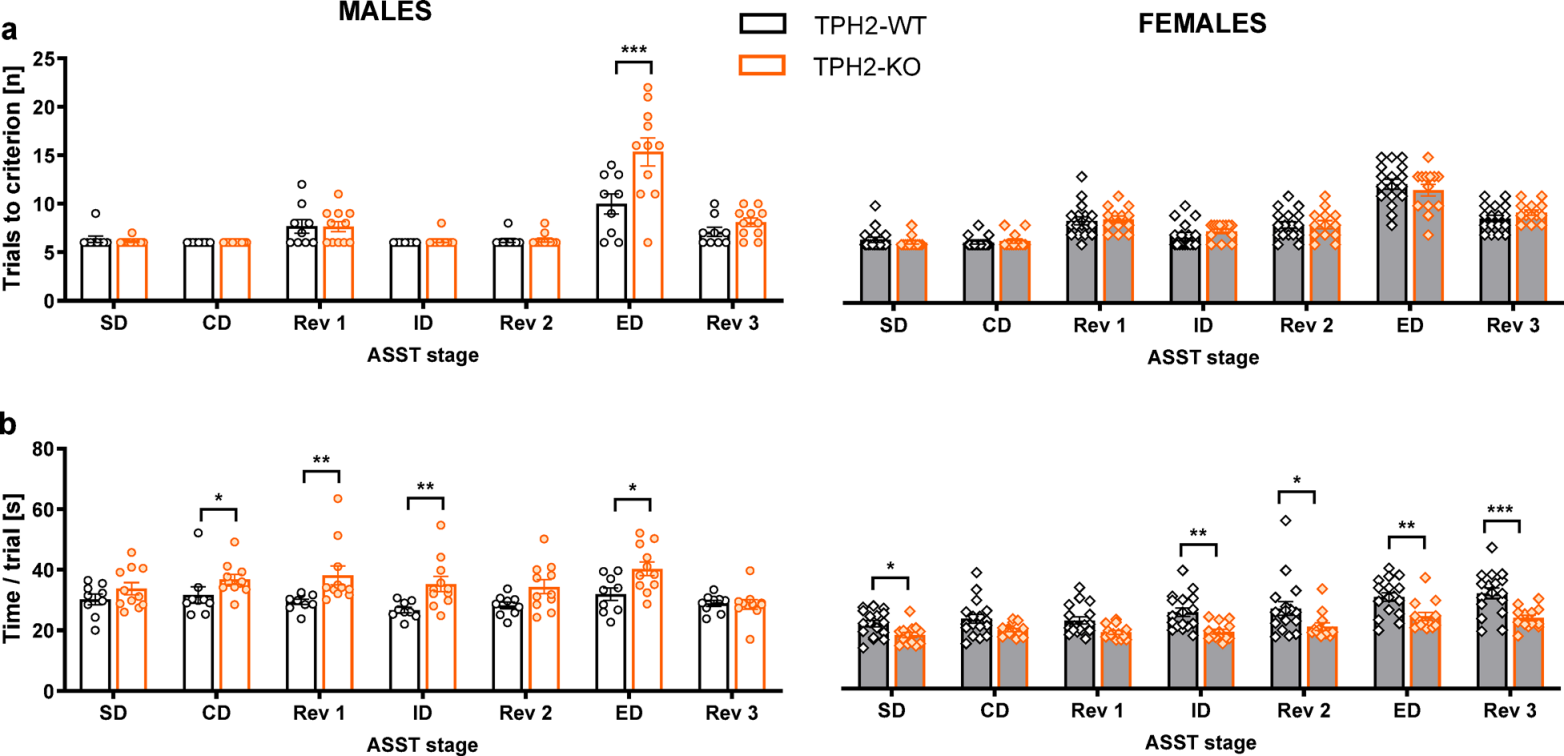
TPH2 deletion induces cognitive inflexibility in male rats. Cognitive flexibility was evaluated in the Attentional Set Shifting Task (ASST). On the last day of the test, the rats performed a series of 7 discriminations in a single test session: simple discrimination (SD), compound discrimination (CD), reversal 1 (Rev1), intradimensional shift (ID), reversal 2 (Rev2), extradimensional shift (ED), and reversal 3 (Rev3). Data are presented as mean ± SEM of trials to criterion (**a**) and the time to complete a single trial during a given task stage (**b**). Symbols: *p < 0.05, **p < 0.01, ***p < 0.001, a significant difference between TPH2-WT and TPH2-KO animals in a given sex (planned comparisons).

TPH2 deletion affected rats’ set-shifting performance in a sex-specific manner (genotype × sex × test phase interactions for the TTC measure: F[2.6,119.71] = 6.51, *p* < 0.001; Fig. 7a, Table S.3.6 Supplement 3). Specifically, TPH2-KO males showed cognitive inflexibility, requiring more trials to complete the extradimensional shift stage of the task (t = 3.88, *p* < 0.001). There was no effect of genotype during any other task stages. No differences were observed between TPH2-WT and TPH2-KO females in the TTC measure across any ASST phase.

Moreover, TPH2-KO males required more time to complete the trial than their WT counterparts (*p* < 0.01, Tukey HSD post hoc test following a significant genotype x sex interaction: F[1,46] = 27.67, *p* < 0.001; Fig. 7b, Table S.3.6 Supplement 3). Planned comparisons revealed significant differences between TPH2-WT and TPH2-KO males at the CD (t = 2.05, *p* < 0.05), Rev1 (t = 3.36, *p* < 0.001), ID (t = 3.48, *p* < 0.001), and ED (t = 3.04, *p* < 0.01) stages. In contrast, TPH2-KO females completed the trials faster than their WT counterparts (*p* < 0.001, Tukey HSD post hoc test; Fig. 7b). These reductions were observed at the SD (t = 2.11, *p* < 0.05), ID (t = 3.15, *p* < 0.01), Rev2 (t = 2.28, *p* < 0.05), ED (t = 2.83, *p* < 0.01), and Rev3 (t = 4.18, *p* < 0.001) stages. The oestrous cycle did not influence females’ performance on the ASST (Fig. S.2.4, Table S.2.5 Supplement 2).

## Discussion

Autism, as a disorder with unknown aetiology, may result from genetic mutations affecting the serotonergic system^10,49^. Building on previous scientific studies in mice^14,15,50^, we examined behavioural phenotypes of TPH2-KO rats to evaluate their potential as a serotonin-depleted autism model. All tests were performed in the context of core autism symptoms, with a particular emphasis on social communication deficits. The present study demonstrated that constitutive depletion of TPH2 in the brain impairs socio-communicative abilities and induces repetitive behaviours (see summary in Table S.5.1 Supplement 5).

Starting with the impact on social interactions, there are distinct behaviour patterns in 5-HT-deficient animals compared to controls. Both male and female TPH2-KO rats exhibited reduced climbing and following behaviours. Examining the females’ results more closely reveals that not only were climbing and following behaviours reduced, but the number of sniffing and anogenital sniffing episodes also decreased. Despite the challenges in assessing reciprocal social interactions in TPH2-KO mice, where heightened aggression is a confounding factor, some studies have nonetheless reported reduced social contact in both juvenile and adult mutants^14,15^. Moreover, beyond increased aggression, home cage monitoring of TPH2-KO mice under semi-natural conditions has revealed a broader maladaptive phenotype that may underlie disruptions in social network structure and group formation dynamics^51^. In addition, TPH2 deletion impairs the processing of social olfactory cues, resulting in deficits in social recognition^14,51,52^.

In line with the well-established link between low brain serotonin and aggression, male TPH2-KO rats’ social interactions included aggressive behaviours. This tendency was further confirmed by the resident-intruder test results, in which both male and female TPH2-KO rats exhibited increased episodes of dominant behaviour. These findings align with previous studies in TPH2-deficient mice^15,24,53^ and rats^36,37,54^, indicating that rodents with brain serotonin depletion exhibit not only impaired social behaviour but also heightened aggression. Additionally, a recent study revealed that even heterozygous TPH2 female mice may exhibit heightened aggression when exposed to stress and food deprivation^55^. A similar effect has been observed in humans, where serotonin deficiency can trigger aggressive behaviour^56^. For instance, individuals on a low-tryptophan diet, which reduces serotonin levels, exhibit increased aggression—a response that is reversible with SSRIs^57,58^. It has also been shown that low cerebrospinal 5-HIAA levels correlate with elevated aggression in humans^59^. Additionally, TPH2 polymorphisms are linked to functional and neuronal changes associated with aggression^57^. Notably, aggression is prevalent in a subset of individuals with ASD^60^ and has also been observed in animal models of the condition^61^. Therefore, the current findings further support the presence of an autistic-like phenotype in TPH2-KO animals.

Among the various mechanisms regulating 5-HT neurotransmission, inhibitory 5-HT_1A_ autoreceptors have been implicated in animal studies as key modulators of aggressive behaviour and mediators of the robust anti-aggressive effects of 5-HT_1A_ receptor agonists^62^. In this context, the heightened aggressiveness observed in TPH2-KO rats may be partly attributable to a reduced sensitivity of 5-HT_1A_ receptors. This interpretation is supported by the rightward shift observed in the dose-response curve for the anti-aggressive effects of the 5-HT_1A_ receptor agonist NLX-112 in these animals compared to their wild-type counterparts^63^. Notably, this finding contrasts with previous reports in TPH2-KO mice, where enhanced 5-HT_1A_ autoreceptor function was observed^64^. These discrepancies suggest that, in the absence of 5-HT, 5-HT_1A_-mediated regulation of aggression may involve more complex mechanisms, potentially including a contribution of postsynaptic 5-HT_1A_ receptors.

An alternative hypothesis to explain the differential behaviour patterns and increased aggression in TPH2-KO rats is altered oxytocin signalling, potentially arising as a compensatory mechanism in these rats. In TPH2-KO rats, both oxytocin and its receptor were found to be more abundant compared to WT controls, potentially influencing the regulation of aggression and social behaviour^36^. Studies in both humans and rodents indicate that oxytocin’s effects on social behaviour are gender-dependent^65^. This gender-specific action of oxytocin might explain the behavioural differences observed between males and females, particularly the increased aggression in males during both the social interaction and resident-intruder tests.

Another potential factor underlying the differences in behavioural patterns could be testosterone. Present in higher concentrations in males than in females, this hormone is linked to the regulation of social aggression in both humans and animals. Studies suggest a relationship between testosterone levels and brain serotonin in modulating aggression^56,66^. Testosterone impacts the brain serotonergic system by influencing cerebral 5-HT metabolism^67^. Additionally, the role of cortisol in aggressive behaviour is well-documented: elevated testosterone and reduced cortisol, combined with low brain serotonin, may contribute to aggressive tendencies^56,66^. Moreover, elevated corticosterone levels were reported in adult TPH2-KO male rats^37^. These findings support an aggressive phenotype associated with TPH2 deletion and may help explain the observed gender differences in aggression between male and female TPH2-KO rats.

USV, a measure of rodent communication, serves as an effective marker for identifying socio-communicative deficits in preclinical studies^25,68^. Given rats’ rich acoustic communication system and their complex behavioural repertoire, USVs offer valuable insight into autism-related socio-communicative deficits. Despite this, studies specifically addressing ultrasonic abnormalities in TPH2-KO animals are scarce and mostly focus on evaluating USVs during maternal separation in pups^69,70^ or juvenile mice^15^. In our study, we recorded and analysed USVs in rats during social interaction and resident-intruder tests. TPH2-KO rats demonstrated distinct patterns of USVs compared to WT controls, supporting the presence of communication deficits in TPH2-KO rats, which aligns with the core symptoms of autism.

Our study demonstrated that TPH2 deficiency affects USV emission in a sex-specific manner during both the social interaction and resident-intruder tests. Female TPH2-KO rats vocalised less during social interactions, although the acoustic properties of their calls remained unchanged. In contrast, male TPH2-KO rats exhibited more pronounced changes in both the quantity and acoustic quality of their calls. They emitted more USVs compared to WT controls, and their calls were longer (increased average duration), wider (greater bandwidth), and emitted on a higher peak frequency. This pattern of changes was consistent across the social interaction and resident-intruder tests.

Interestingly, a positive correlation was observed between the number of 50-kHz vocalizations emitted by TPH2-KO males and the expression of specific behaviours. In particular, the emission of 50-kHz calls increased in parallel with aggressive and copulatory-like behaviours. A more detailed analysis revealed that trill-type calls were particularly associated with aggressive encounters. These findings suggest that the elevated vocal output in TPH2-KO males, relative to females, may be partially attributable to their increased expression of these behaviours. Notably, 50-kHz calls are not exclusively linked to positive affective states, as they have also been reported during aggressive behaviour^71^. This suggests that the increased vocal activity in TPH2-KO males likely reflects a heightened level of social arousal or engagement, particularly in dominance-related behaviours. Moreover, these vocalizations are considered indicators of affective state^26^ and have been shown to emerge in response to social stress^25^. These findings suggest that the heightened calling observed in TPH2-KO males may represent a behavioural manifestation of altered emotional reactivity and social coping mechanisms associated with serotonin deficiency. It is important to note that aggression is not always associated with violence but can serve as a highly functional form of social communication^22^. In support of this hypothesis, during the social interaction test, TPH2-KO rats did not show a significant increase in aversive 22-kHz sounds, which are typically associated with aggression and fighting behaviour^68^.

Repetitive behaviours can be categorised into two clusters: less complex actions, such as object manipulation, stereotyped movements or self-injurious actions, and more complex behaviours that involve cognitive components, including compulsions and rituals. In rodents, motor behaviours can be assessed using the marble burying test. More complex forms of stereotypes, which often manifest as cognitive inflexibility or rigidity, traits commonly associated with autism, can be evaluated in rodents using the ASST^34,72^.

Our findings indicate that brain serotonin depletion in rats contributes to repetitive behaviour. While male TPH2-KO rats tended to increase the number of buried marbles, females demonstrated a significant increase in compulsivity in this test. Our results align with other researchers’ results, showing increased marble-burying in TPH2-KO mice^6,14,50^ and rats^54^. The studies in TPH2-KO rodents align with reports demonstrating that long-term or short-term tryptophan depletion, as well as perinatal SSRI administration, leads to the development of repetitive behaviours characteristic of autism^4,73^.

Moreover, TPH2 deficiency also led to cognitive inflexibility, manifested by an increased number of trials to criterion during the ED stage of the ASST. Interestingly, this rigidity in cognitive abilities was observed only in males. To our knowledge, there is currently limited information on how TPH2 deficiency affects ASST performance, particularly in a sex-specific manner, highlighting the novelty of these findings. Nevertheless, TPH2 mutant mice have previously demonstrated impaired reversal learning in a maze-based task^74^. This finding aligns with the well-established role of serotonin in regulating reversal learning, as shown in humans following acute tryptophan depletion^75^ and in marmosets with prefrontal serotonin depletion^76,77^. However, these studies also indicate that while serotonin is critical for flexible responding at the level of changing stimulus-reward contingencies, it may not be essential for the higher-order shifting of an attentional set. Consequently, the ED shift impairments observed in TPH2-KO males may reflect not only the consequences of serotonin depletion per se, but also secondary neurodevelopmental adaptations or compensatory mechanisms that arise from the lifelong absence of central serotonin.

Limitations. Although TPH2-KO rats exhibit phenotypes mimicking certain autism-related behaviours, the extent to which these findings translate to human autism should be interpreted cautiously. While animal models provide valuable insights, their relevance to human neuropsychiatric conditions is limited by aetiological heterogeneity and variability in symptom manifestations in ASD. Moreover, the behavioural tests used may not fully capture the complexity of autism-related behaviours in humans, as they tend to oversimplify the diverse range of repetitive and socio-communicative deficits characteristic of the disorder. On the other hand, it cannot be excluded that a more detailed examination, such as temporal pattern analysis, could yield deeper insights into the organization and dynamic structure of behavioural patterns. Additionally, the assessment primarily focused on core autism symptoms, but it did not address other dimensions such as sensory sensitivity or comorbid conditions like anxiety and hyperactivity, which may also shape the phenotype. While the study identified sex differences, their underlying mechanisms warrant further investigation. Finally, it is important to recognize that ASD is highly heterogeneous, arising from a complex interplay of genetic and environmental factors. Further research is needed to explore how environmental factors influence the phenotype in TPH2-KO animals, shedding light on the role of TPH2 in ASD aetiology.

In summary, central serotonin deficiency impairs socio-communicative abilities and promotes motor and cognitive repetitive behaviours. Notably, the sex differences observed in the TPH2-KO rats suggest that males and females may be affected differently, underscoring the importance of considering sex as a variable in neuropsychiatric research. The TPH2-KO rat may thus serve as a valuable tool for investigating the role of serotonin in behavioural disturbances associated with neuropsychiatric disorders, such as autism.

## Electronic supplementary material

Below is the link to the electronic supplementary material.

Supplementary Material 1

## Data Availability

All data generated during this study are included in this article and its supplementary file.
