# Supplementary material for: Tryptophan hydroxylase 2 deficiency alters autism-related behavioural phenotypes in rats
Source: Sci Rep. 2025 Jul 1;15:20522. doi: 10.1038/s41598-025-05684-9 (PMC12215488; doi:10.1038/s41598-025-05684-9)
Supplement: Supplementary file 1 — Supplementary Material 1 [file 41598_2025_5684_MOESM1_ESM.pdf]

Supplementary materials

Tryptophan hydroxylase 2 deficiency alters autism-related behavioural phenotypes in rats

Joanna Golebiowska [1], Małgorzata Holuj [1], Natalia Alenina [2,3] , Michael Bader [2-5], Piotr Popik [1], Agnieszka Nikiforuk [1*]

Supplement 1

The attentional set-shifting task (ASST) procedure

The ASST evaluates cognitive flexibility, a component of executive control subserved by the prefrontal cortex (Birrell & Brown, 2000).

As previously described (e.g., Popik & Nikiforuk, 2015; Potasiewicz et al., 2020), testing was conducted in a dimly illuminated (20 Lux) Plexiglas apparatus (length x width x height: 38 x 38 x 17 cm) with the grid floor and wall dividing half of the length of the cage into two sections. During testing, one glass digging pot (internal diameter of 6 cm and a depth of 3 cm) was placed in each section. Each pot was defined by a pair of stimulus cues with two stimulus dimensions (odour and digging medium). To mark each pot with a distinct odour, 5 µl of a flavouring essence (Dr. Oetker®, Poland or The Body Shop, UK) was applied to a piece of blotting paper fixed to the external rim of the pot immediately before use. A different pot was used for each combination of digging medium and odour; only one odour was ever applied to a given pot. The bait (one-half of a Honey Nut Cheerio, Nestle®) was placed at the bottom of the "positive" pot and buried in the digging medium. A small amount of powdered Cheerio was added to the digging media to prevent the rat from trying to detect the buried reward by its smell.

The procedure was conducted over a period of three consecutive days for each rat.

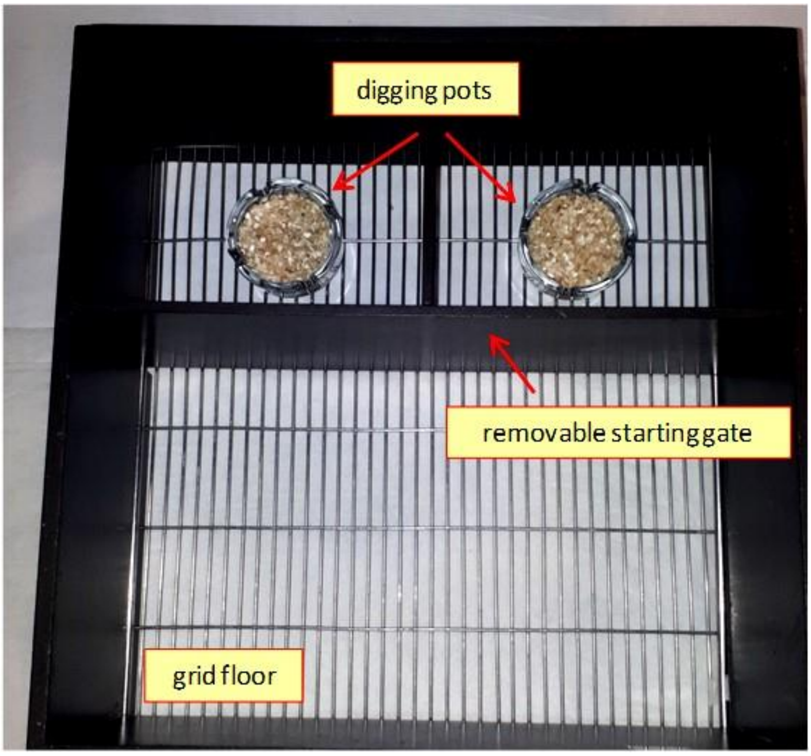

Fig.S.1.1. Plexiglas ASST apparatus.

Day 1. Habituation

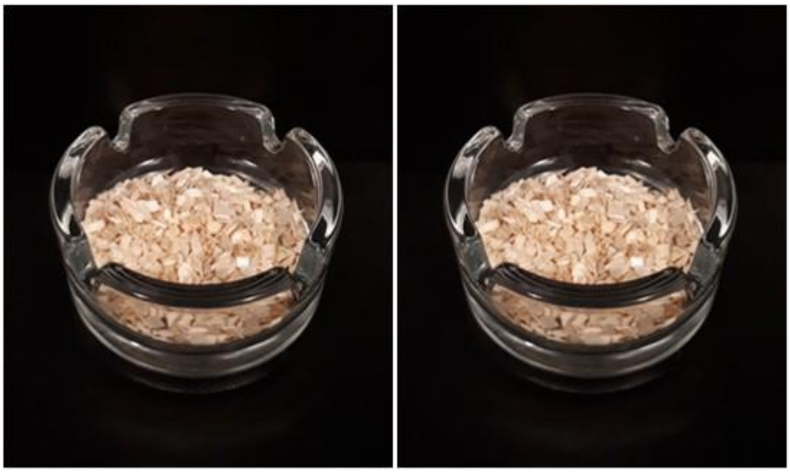

Fig.S.1.2. Example of bowl sawdust-filled.

Rats were habituated to the testing area and trained to dig in pots filled with sawdust to retrieve the food reward.

The rats were transported from the housing facility to the testing room, where they were presented with one unscented pot (filled with several pieces of Cheerios) in their home cages. After the rats had eaten the Cheerio from the home cage pot, they were placed in the apparatus and given three trials to retrieve the reward from both sawdust-filled baited pots. With each exposure, the bait was covered with an increasing amount of sawdust. Animals that did not dig for a food reward over 3 consecutive daily sessions were excluded from the experiment.

Day 2. Training

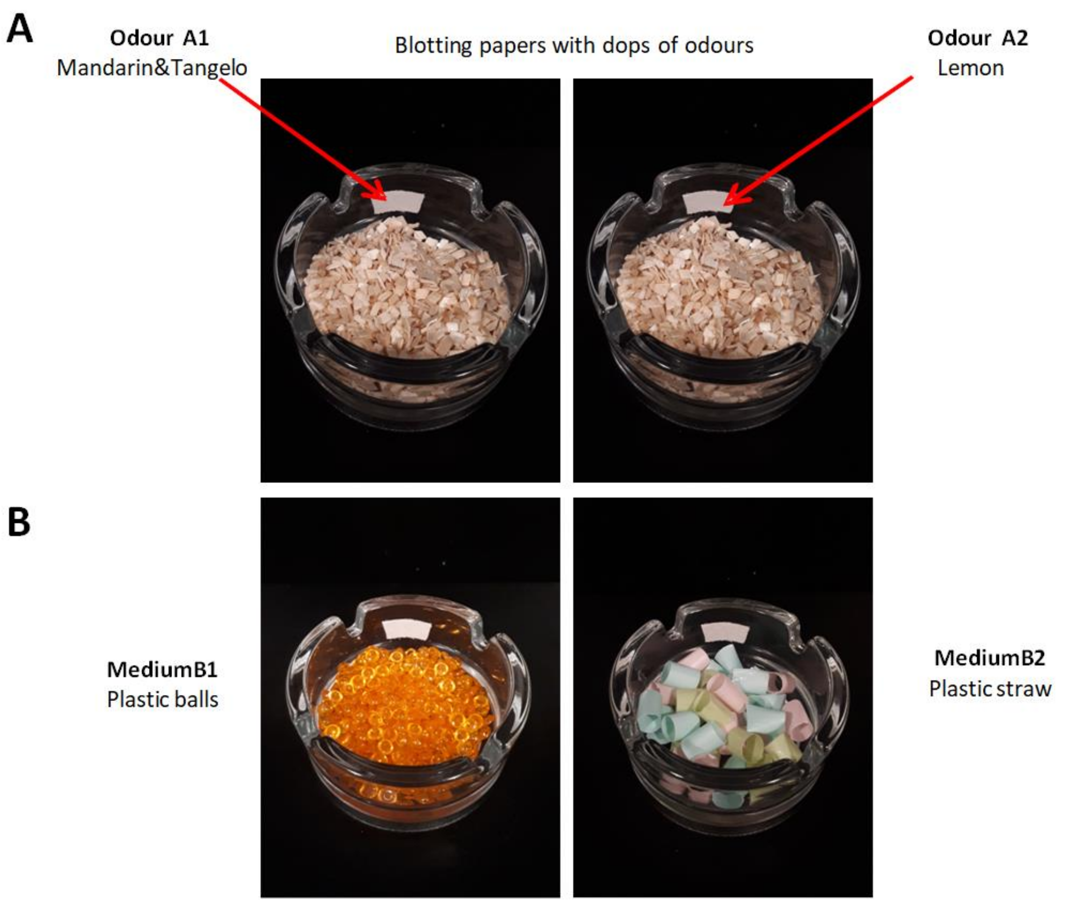

Fig.S.1.3. Examples of stimuli used in the ASST training phase: (A) Example of odour (i.e. flavour essences) placement and (B) Example of media placed bowls.

Rats were trained on a series of simple discriminations (SDs) to a criterion of six consecutive correct trials.

For these trials, the rats had to learn to associate the food reward with an odour cue (e.g., arrack vs. orange, both pots filled with sawdust) or a digging medium (e.g., plastic balls vs. pebbles, no odour). All rats were trained using the same pairs of stimuli. The positive and negative cues for each rat were presented pseudo-randomly and equally. These training stimuli were not used again in later testing trials.

Day 3.Testing

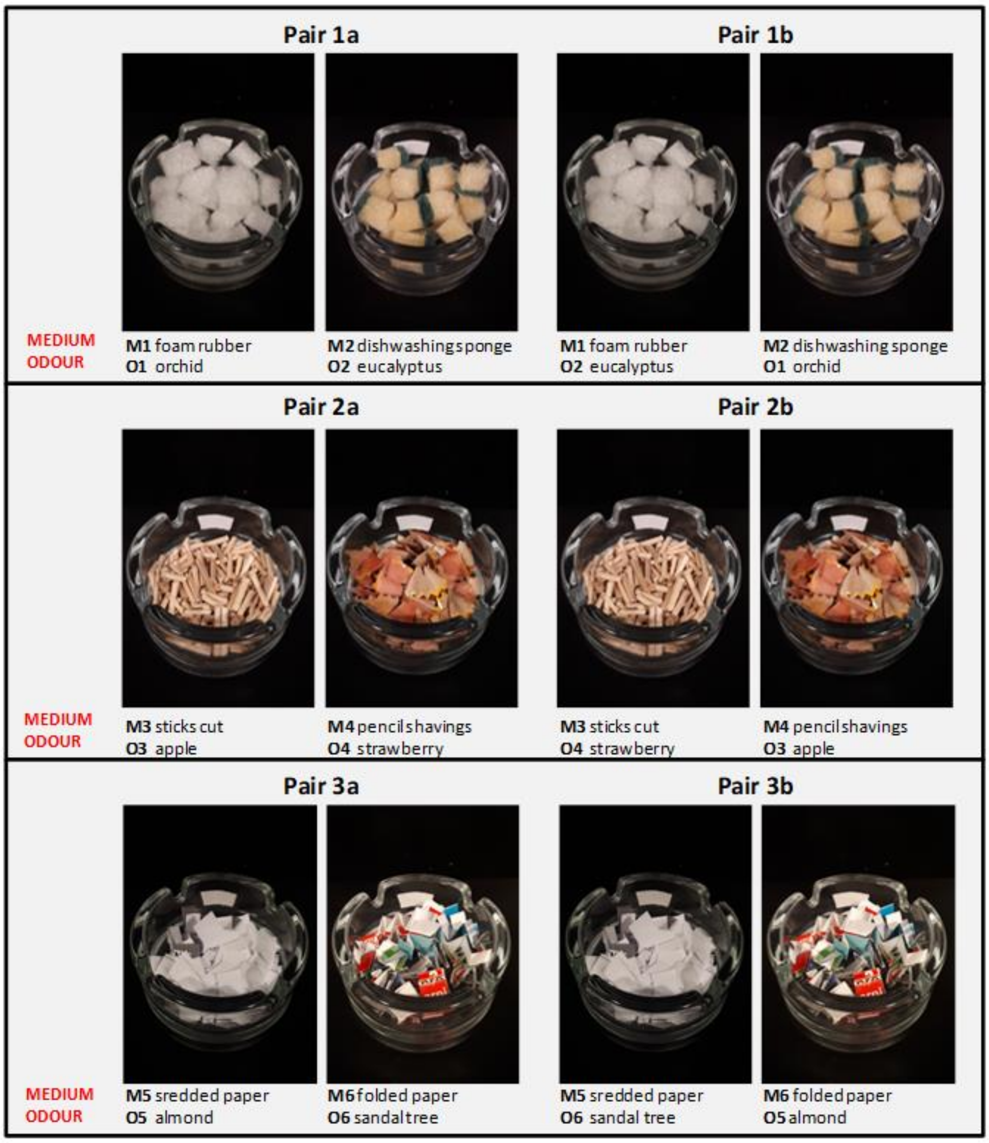

Fig.S.1.4. Examples of stimuli pairs used in the ASST testing phase.

Rats performed a series of discriminations in a single test session.

The first trial at the beginning of each discrimination phase was a discovery trial, during which the animals were allowed to dig in both bowls; this trial counted toward the six criterion trial. In the subsequent trials, each incorrect choice was recorded as an error. Digging was defined as any distinct displacement of the digging media with either the paw or the nose; the rat could investigate a digging pot by sniffing or touching it without displacing material. Testing was continued at each phase until the rat reached the criterion of six consecutive correct trials, after which testing proceeded to the next phase.

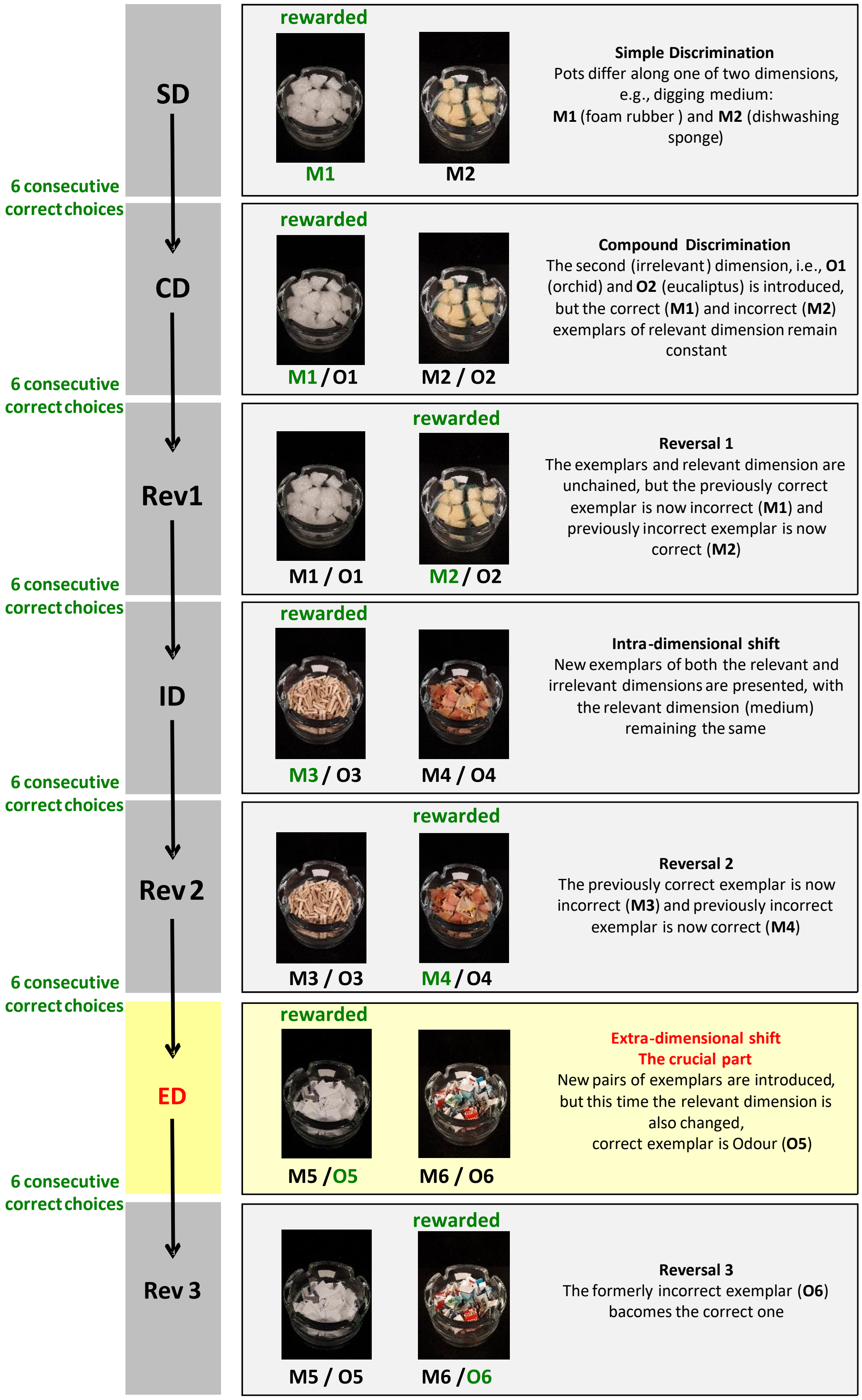

Fig. S.1.5. Diagram of the ASST Testing Phase

In the simple discrimination involving only one stimulus dimension, the pots differed along one of the two dimensions (e.g., digging medium). For the compound discrimination (CD), the second (irrelevant) dimension (i.e., odour) was introduced, but the correct and incorrect exemplars of the relevant dimension remained constant. The exemplars and the relevant dimension were unchanged for the reversal of this discrimination (Rev 1), but the previously correct exemplar was now incorrect, and vice versa. The intra-dimensional (ID) shift was then presented, comprising new exemplars of both the relevant and irrelevant dimensions, with the relevant dimension remaining the same as previously described. The ID discrimination was then reversed (Rev 2) so that the formerly positive exemplar became the negative one. For the extra-dimensional (ED) shift, a new pair of exemplars was again introduced; however, this time, the relevant dimension was also changed. Finally, the last phase was the reversal (Rev 3) of the ED discrimination.

The exemplars were always presented in pairs, and they varied so that only one animal within each treatment group received the same combination. Each exemplar's assignment in a pair as positive or negative at a given phase and the pots' left-right positioning in the test apparatus on each trial were randomized.

Supplement 2.

The effect of the oestrous cycle on females' performance.

Determination of the estrus cycle

Rat's oestrous cycle was divided into 4 phases: proestrus, estrus, metestrus and diestrus. The current stage of the cycle was determined by the results of vaginal cytology. Smear samples were collected 2 hours after the end of the test using sterile cotton-tipped swabs. The swabs were gently and shallowly inserted into the vaginal orifice (approximately 1 cm) to avoid overstimulation that could lead to pseudopregnancy. After gently rotating the swab against the vaginal wall (2-3 twists) to collect vaginal epithelial cells, the samples were prepared on glass slides. Using a light microscope with a 20× objective lens, the oestrous cycle stage was determined based on the appearance and abundance of cells within vaginal samples. When the sample examination showed a low concentration of leukocytes and a high concentration of cells of polygonal shape and small nuclei, the stage was identified as proestrus. Estrus was characterized by the presence of a large number of cells with enucleated and needle-like shapes or clumps of rounded cells with jagged edges. In metestrus, a combination of leukocytes and clusters of rounded, nucleated epithelial cells was observed. Diestrus was identified by a large number of leukocytes and a few small clumps of nucleated epithelial cells (Potasiewicz et al., 2020; Schneider & Popik, 2009).

The effect of the oestrous cycle on social behaviours in female rats

Data on separate behaviours were analyzed by a repeated measure ANOVA with cycle stage (metestrus - diestrus and proestrus - estrus) and genotype as between-subject factors and the type of behaviour as a repeated measure. There were no significant effects of the oestrous cycle and cycle × genotype or cycle × genotype × behaviour interactions (Fig. S.2.1, Table S.2.2). Tukey post hoc test conducted after a significant cycle × behaviour interaction revealed that more sniffing episodes were recorded during the proestrus – estrus phase ($p<0.001$).

The total number of episodes was subjected to a two-way ANOVA with the cycle stage and genotype as between-subject factors (Table S.2.2).

Fig. S.2.1. The effect of the oestrous cycle on social behaviour in TPH2-WT and TPH2-KO females.

Table S.2.1. Separate behaviors					
	df1	df2	F	p	η_p^2
Genotype (G)	1	48	26.935	<0.001	0.359
Cycle phase (C)	1	48	2.715	0.106	0.054
G×C	1	48	0.202	0.655	0.004
Behaviour (B)	2.33	111.68	388.605	<0.001	0.890
B×G	2.33	111.68	12.159	<0.001	0.202
B×C	2.33	111.68	6.106	0.0018	0.113
B×C×G	2.33	111.68	0.513	0.6275	0.011

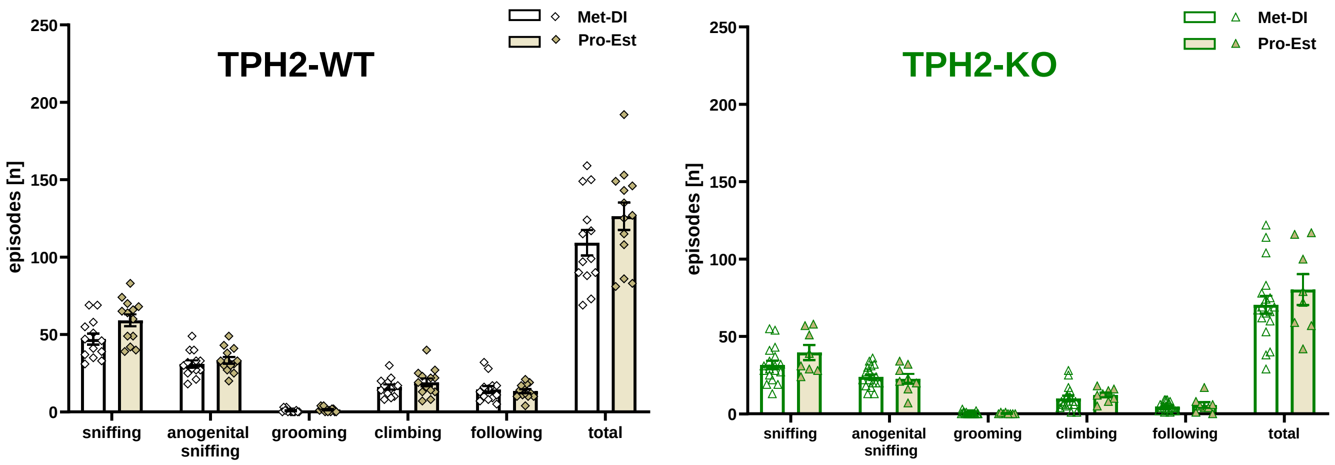

Table S.2.2. Total number of SI episodes					
	df1	df2	F	p	η_p^2
Genotype (G)	1	48	26.935	<0.001	0.359
Cycle phase (C)	1	48	2.715	0.106	0.054
G×C	1	48	0.202	0.655	0.004

The effect of the oestrous cycle on females' behaviour in the resident-intruder test

Data were analyzed using a repeated measure ANOVA with the cycle stage (metestrus - diestrus and proestrus - estrus) and genotype as between-subject factors and the type of behaviour (dominant vs. submissive) as a repeated measure. There were no significant effects of the oestrous cycle and cycle × genotype or cycle × genotype × behaviour interactions (Fig. S.2.2, Table S.2.3).

Fig.S.2.2. The effect of the oestrous cycle on females' behaviour in the resident-intruder test.

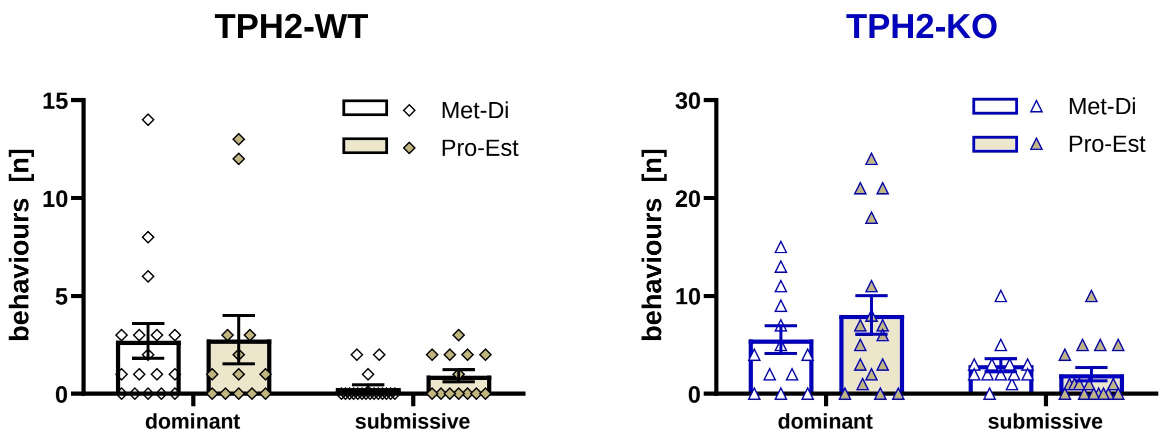

Table S.2.3. Resident - Intruder					
	df1	df2	F	p	η_p^2
Genotype (G)	1	56	11.861	0.001	0.175
Cycle phase (c)	1	56	0.444	0.508	0.008
G×C	1	56	0.069	0.793	0.001
Behaviour (B)	1	56	21.165	<0.001	0.274
B×G	1	56	2.468	0.122	0.042
B×C	1	56	1.048	0.310	0.018
B×C×G	1	56	2.034	0.159	0.035

Effect of the oestrous cycle on marble burying activity of female rats.

The percentage of buried marbles (Fig. S.2.3a) and distance travelled (Fig. S.2.3b) were analyzed by a two-way ANOVA with the cycle stage and genotype as between-subject factors.

Fig. S.2.3. Effect of the oestrous cycle on marble burying activity in TPH2-WT and TPH2-KO females.

Table S.2.4. marble-burying test								
	Buried marbles [n]					Distance traveled [m]		
	df1	df2	F	p	η_p^2	F	p	η_p^2
Genotype (G)	1	35	3.344	0.0759	0.087	18.269	<0.001	0.343
Cycle (C)	1	35	0.438	0.5124	0.012	0.326	0.572	0.009
G×C	1	35	0.773	0.3854	0.022	0.077	0.784	0.002

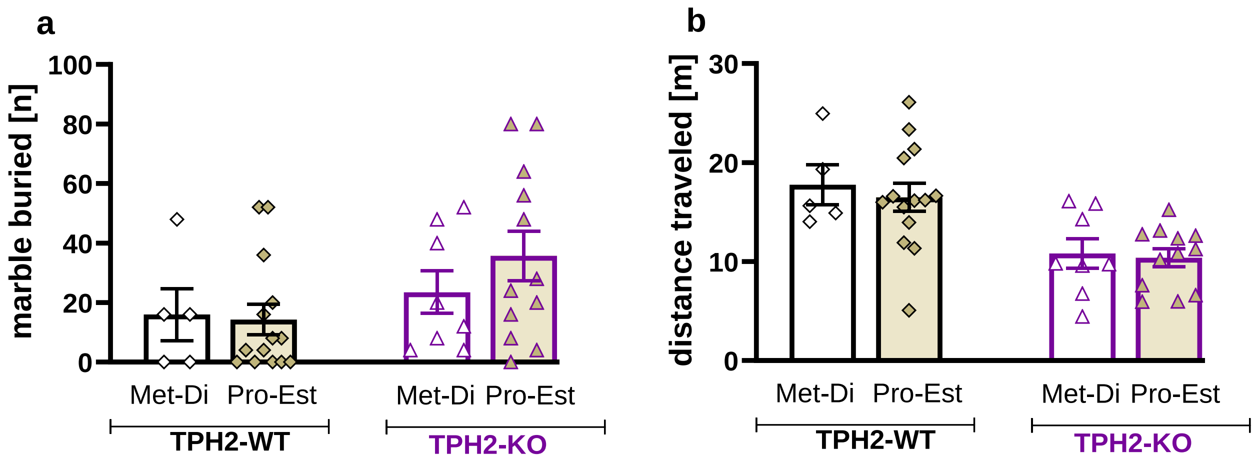

Effect of the oestrous cycle on ASST performance in female rats

Data were analyzed by a repeated measure ANOVA with cycle stage (metestrus - diestrus and proestrus - estrus) and genotype as between-subject factors and the ASST phase as a repeated measure.

Fig.S.2.4. Effect of the oestrous cycle on ASST performance in TPH2-WT and TPH2-KO females

Table S.2.5. ASST

Effect	df1	df2	TTC			Time/Trial		
			F	p	η_p^2	F	p	η_p^2
Genotype (G)	1	26	0.069	0.794	0.003	16.448	<0.001	0.387
Cycle phase (C)	1	26	0.014	0.907	0.001	0.386	0.539	0.015
G×C	1	26	1.924	0.177	0.069	0.093	0.763	0.004
ASST phase (P)	3.79	98.49	63.96	<0.001	0.711	15.539	<0.001	0.374
P×G	3.79	98.49	0.731	0.566	0.027	1.329	0.247	0.049
P×C	3.79	98.49	0.469	0.748	0.018	1.256	0.281	0.046
P×C×G	3.79	98.49	1.432	0.231	0.052	0.558	0.764	0.021

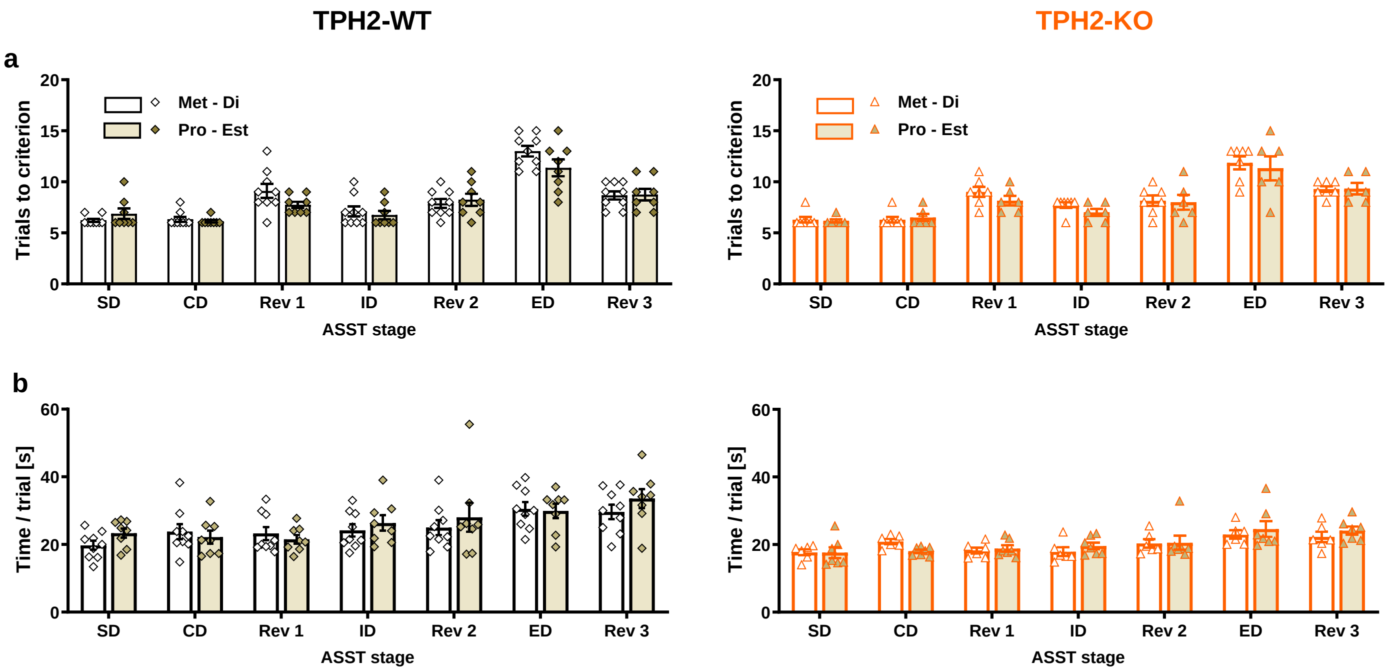

Supplement 3

Details of the ANOVA analysis.

Table S.3.1. Social Interaction (SI) test					
**Effect**	**df1**	**df2**	**F**	**p**	η_p^2
**Genotype (G)**	1	55	17.411	<0.001	0.240
**Sex (S)**	1	55	0.192	0.663	0.003
**G×S**	1	55	48.833	<0.001	0.470
**Behavior (B)**	2.75	151.37	607.529	<0.001	0.917
**BxG**	2.75	151.37	18.677	<0.001	0.254
**B×S**	2.75	151.37	21.624	<0.001	0.282
**B×T×S**	2.75	151.37	11.475	<0.001	0.173

Table S.3.2. SI: ultrasonic vocalizations														
**Effect**	**df1**	**df2**	**USV number**			**duration**			**bandwidth**			**peak frequency**		
			**F**	**p**	η_p^2	**F**	**p**	η_p^2	**F**	**p**	η_p^2	**F**	**p**	η_p^2
**Genotype (G)**	1	55	0.352	0.555	0.006	1.890	0.175	0.033	17.658	<0.001	0.243	36.612	<0.001	0.399
**Sex (S)**	1	55	20.852	<0.001	0.275	5.744	0.019	0.094	100.903	<0.001	0.647	26.357	<0.001	0.323
**G×S**	1	55	30.518	<0.001	0.358	3.169	0.081	0.054	34.373	<0.001	0.385	48.030	<0.001	0.466

Table S.3.3. Resident-Intruder (R-I) test					
**Effect**	**df1**	**df2**	**F**	**p**	η_p^2
**Genotype (G)**	1	91	21.074	<0.001	0.188
**Sex (S)**	1	91	1.039	0.311	0.011
**G×S**	1	91	0.111	0.739	0.0012
**Behavior (B)**	1	91	51.011	<0.001	0.359
**BxG**	1	91	16.594	<0.001	0.154
**B×S**	1	91	3.345	0.071	0.305
**B×T×S**	1	91	4.751	0.032	0.049

Table S.3.4. R-I: ultrasonic vocalizations														
**Effect**	**df1**	**df2**	**USV number**			**duration**			**bandwidth**			**peak frequency**		
			**F**	**p**	η_p^2	**F**	**p**	η_p^2	**F**	**p**	η_p^2	**F**	**p**	η_p^2
**Genotype (G)**	1	91	7.101	0.009	0.072	34.127	<0.001	0.273	50.482	<0.001	0.357	0.052	0.820	0.001
**Sex (S)**	1	91	5.668	0.019	0.059	18.338	<0.001	0.168	45.549	<0.001	0.334	6.335	0.014	0.065
**G×S**	1	91	1.347	0.249	0.015	21.904	<0.001	0.194	12.484	<0.001	0.121	0.947	0.333	0.011

Table S.3.5. marble burying test								
			Buried marbles [n]			Distance traveled [m]		
	df1	df2	F	p	η_p^2	F	p	η_p^2
Genotype (G)	1	66	7.426	0.008	0.101	45.278	<0.001	0.407
Sex (S)	1	66	2.498	0.119	0.036	5.378	0.023	0.075
G×S	1	66	0.359	0.551	0.005	0.044	0.834	0.0001

Table S.3.6. ASST										
Effect	*TTC*					Time/Trial				
	df1	df2	F	p	η_p^2	df1	df2	F	p	η_p^2
Genotype (G)	1	46	6.092	0.017	0.117	1	46	0.017	0.898	0.000
Sex (S)	1	46	12.931	<0.001	0.219	1	46	78.445	<0.001	0.630
G×S	1	46	4.609	0.037	0.091	1	46	27.667	<0.001	0.376
ASST phase (P)	2.6	119.71	91.408	<0.001	0.665	4.6	211.63	6.987	<0.001	0.132
P×G	2.6	119.71	4.016	0.013	0.080	4.6	211.63	2.259	0.055	0.047
P×S	2.6	119.71	3.745	0.017	0.075	4.6	211.63	8.899	<0.001	0.162
P×G×S	2.6	119.71	6.505	<0.001	0.124	4.6	211.63	1.323	0.259	0.028

Supplement 4

USVs emission during the social interaction and resident-intruder tests.

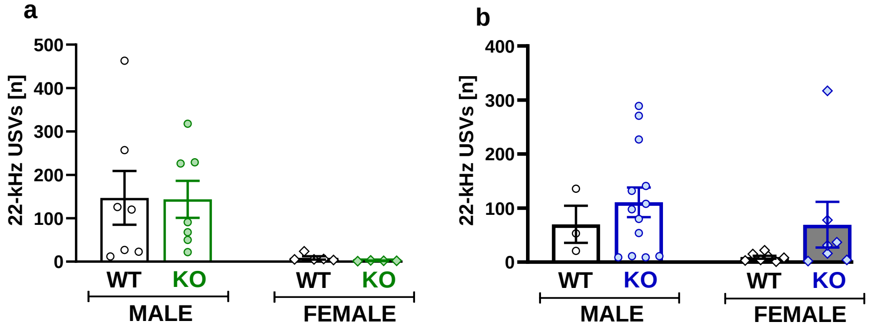

Fig.S.4.1. 22-kHz (alarm) USV emission in TPH2-WT and TPH2-KO rats during the social interaction (a) and resident-intruder (b) tests. Data are presented as mean ± SEM of the total number of alarm calls. The figure includes only alarm-emitting rats (details in the result description).

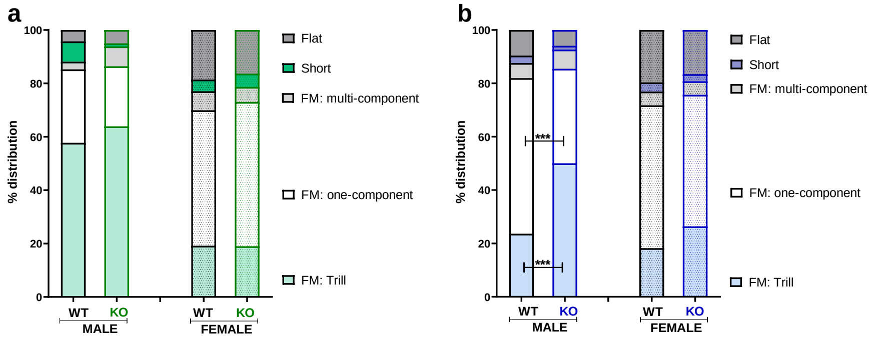

Fig. S.4.2. The distribution of 50-kHz USV types emitted during the social interaction (a) and resident-intruder (b) tests. Data are presented as the percentage distribution of call categories. Symbols: ***p<0.001, a significant difference between TPH2-WT and TPH2-KO animals in a given sex (planned comparisons).

Table S.4.1. SI: percentage USV distribution					
**Effect**	**df1**	**df2**	**F**	**p**	**η_p^2**
**call types (C)**	1.83	100.38	128.519	<0.001	0.700
**C×G**	1.83	100.38	0.625	0.523	0.011
**C×S**	1.83	100.38	72.852	<0.001	0.569
**C×G×S**	1.83	100.38	1.375	0.257	0.024

Table S.4.2. R-I: percentage USV distribution					
**Effect**	**df1**	**df2**	**F**	**p**	**η_p^2**
**call types (C)**	1.65	149.83	240.949	<0.001	0.726
**C×G**	1.65	149.83	20.999	<0.001	0.187
**C×S**	1.65	149.83	13.653	<0.001	0.130
**C×G×S**	1.65	149.83	8.004	0.001	0.081

Supplement 5

Table.S.5.1

			RESULTS	
			**Male** TPK2 KO	**Female** TPH2 KO
	**Social Interaction Test**			
Analysed behaviours	**Sniffing**	the rat sniffs the body of the conspecific	–	↓
	**Anogenital sniffing**	the rat sniffs the conspecific's anogenital region	–	↓
	**Social grooming**	the rat licks and chews the conspecific's fur	–	–
	**Climbing**	the rat climbs over or stands on the conspecific's back	↓	↓
	**Following**	the rat follows the conspecific	↓	↓
	**Aggressive behaviours**	fighting	↑	–
	**Copulatory-like behaviours**		↑	–
	**Resident-Intruder Test**			
Analysed behaviours	**Dominant behaviours**	lateral threatening	↑	↑
		sideway lateral pushing		
		clinch attacking		
		chasing		
		standing on top of the supine intruder		
		aggressive grooming		
		boxing		
		biting		
	**Submissive behaviours**	supine postures	–	↑
		fleeing behaviour		
		defensive upright posture		
		freezing		

References

Birrell, J. M., & Brown, V. J. (2000). Medial frontal cortex mediates perceptual attentional set shifting in the rat. *Journal of Neuroscience*, *20*(11), 4320–4324. https://doi.org/10.1523/jneurosci.20-11-04320.2000

Popik, P., & Nikiforuk, A. (2015). Attentional set-shifting paradigm in the rat. *Current Protocols in Neuroscience*, *2015*, 9.51.1-9.51.13. https://doi.org/10.1002/0471142301.ns0951s72

Potasiewicz, A., Holuj, M., Litwa, E., Gzielo, K., Socha, L., Popik, P., & Nikiforuk, A. (2020). Social dysfunction in the neurodevelopmental model of schizophrenia in male and female rats: Behavioural and biochemical studies. *Neuropharmacology*, *170*. https://doi.org/10.1016/j.neuropharm.2020.108040

Schneider, T., & Popik, P. (2009). An animal model of premenstrual dysphoric disorder sensitive to antidepressants. *Current Protocols in Neuroscience*, (SUPPL. 46), 1–10. https://doi.org/10.1002/0471142301.ns0931s46
